# Acute panuveitis with hypopyon in Crohn's disease secondary to medical therapy: a case report

**DOI:** 10.1186/1752-1947-1-42

**Published:** 2007-07-04

**Authors:** David Haider, Felipe E Dhawahir-Scala, Nicholas G Strouthidis, Nigel Davies

**Affiliations:** 1 Department of Ophthalmology, Chelsea and Westminster Hospital, Fulham Road, London, UK; 2 Manchester Royal Eye Hospital, Oxford Road, Manchester, UK

## Abstract

**Background:**

A case report to highlight the association between rifabutin and hypopyon

**Methods:**

A 56 year old male presented with a one day history of blurred vision in his right eye. He had an established diagnosis of Crohn's disease which was in remission following treatment with rifabutin and clarithromycin. A brisk anterior uveitis with hypopyon and a mild vitritis was detected in the right eye. The acute inflammatory episode resolved following treatment with topical corticosteroids and withdrawal of rifabutin.

**Results:**

The presence of hypopyon is atypical in uveitis associated with inflammatory bowel disease. The association between rifabutin treatment and hypopyon uveitis is well recognised in Mycobacterium avium paratuberculosis. However, use of rifabutin in the management of Crohn's disease is controversial and not widely known to an ophthalmic readership.

**Conclusion:**

This report highlights the importance of keeping abreast of novel therapeutic developments in systemic conditions likely to be encountered in ophthalmology.

## Background

Crohn's disease is a chronic granulomatous inflammatory disease which affects multiple sites throughout the gastro-intestinal system. The ocular manifestations are protean and commonly include episcleritis, scleritis and anterior uveitis[1]. Retinal vasculitis, optic neuropathy and multifocal choroiditis have also been reported [2-4]. In this report we describe a case of panuveitis with hypopyon in a patient with Crohn's disease in whom the presentation was attributable to a therapeutic agent and not the underlying systemic condition.

## Methods

**Figure 1:**
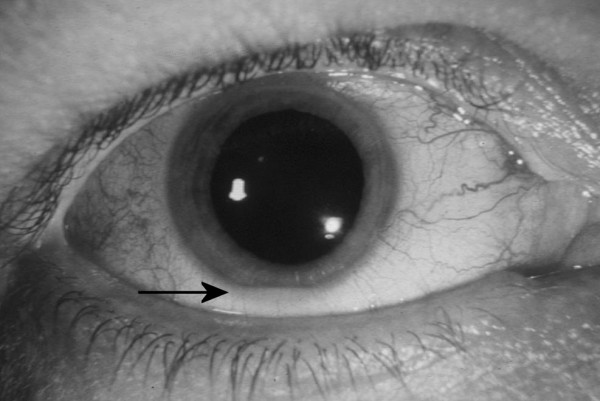
**Hypopyon**. Anterior segment photograph of right eye showing hypopyon at presentation (Arrow pointing at Hypopyon)

A 56 year old male attended the eye clinic having awoken with blurred vision in his right eye; pain and photophobia were not present. A 15 year history of Crohn's was reported – this was in remission following a year-long trial of 300 mg rifabutin daily with clarithromycin 250 mg daily. Azathioprine had been discontinued by his rheumatologist 2 weeks previously. The patient had never undergone gastro-intestinal surgery, there was no arthritis and no previous history of uveitis. He was HLA-B27 negative. At presentation the best corrected visual acuity was 6/24 OD and 6/6 OS. There was a right anterior uveitis with 4 mm hypopyon (Figure 1). The IOP was 12 mmHg in both eyes. There was moderate vitreous activity in the right eye; no features suggestive of retino-choroidal inflammation or vasculitis were detected and the optic disc was healthy. The left eye was quiet, with no evidence of intra-ocular inflammation. Blood tests including a full blood count, erythrocyte sedimentation rate and C-reactive protein were all within the normal range, in keeping with a state of remission.

A diagnosis of right acute panuveitis was made and the patient was commenced on hourly topical dexamethasone 0.1% and cyclopentolate 1% twice daily. The patient discontinued rifabutin, pending rheumatology review the following week. Within three days, the hypopyon had reduced to 1 mm and the vision had improved to 6/9 in the right eye; a reducing course of dexamethasone was instituted. One week after discontinuing rifabutin and clarithromycin, azathioprine 50 mg twice daily was restarted by his rheumatologist. At 4 weeks, the IOP in the right eye had elevated to 38 mmHg – this was ascribed to steroid response and was managed using topical dorzolamide, timolol and by substituting dexamethasone for rimexolone 1% bd. Following a further 8 weeks on this management, the anterior uveitis and ocular hypertension completely resolved and all topical medications were discontinued. At this stage, the patient's inflammatory bowel disease was managed using azathioprine 50 mg twice daily, prednisolone 12.5 mg once a day and alendronate 70 mg once

## Discussion

Acute anterior uveitis occurs in approximately 4% of patients with Crohn's disease. Presentation with hypopyon is unusual and raises suspicion of either alternative systemic inflammatory disorders such as Behçet's or ankylosing spondylitis or, as in this case, an exogenous factor. Hypopyon uveitis is well recognised following rifabutin therapy for mycobacterium avium intracellulare in both immuno-compromised and immuno-competent subjects[5,6]. In these reports, hypopyon could occur bilaterally and usually responded rapidly following commencement of topical corticosteroids, with or without reduction of rifabutin dosage[7]. Presentation with hypopyon often occurs several months after starting rifabutin. In this case the presence of hypopyon, and rapid initial improvement following withdrawal of rifabutin, suggest rifabutin as the underlying aetiology. There is a possibility that the recent discontinuation of azathioprine may have contributed to a generalised inflammatory relapse, of which the uveitis was part of; this was not, however, clinically apparent during the acute presentation. The protracted subsequent recovery is atypical, although the steroid response will undoubtedly have contributed to this.

The use of rifabutin to treat Crohn's disease is not familiar to an ophthalmic readership; a previous case of anterior uveitis has been reported in the general medical literature only[8]. A suspected aetiology of exposure to Mycobacterium avium subspecies has been proposed in Crohn's disease. A particular role for rifabutin has been noted in patients with proven evidence of Mycobacterium avium infection, although this was not the case in this subject[9]. Previous studies reported pharmacokinetic interactions when combining rifabutin and clarithromycin leading to an increase in rifabutin levels, resulting in an increase frequency of uveitis, this could explain the acute onset of uveitis in our case[10].

## Conclusion

The use of rifabutin to treat Crohn's disease is controversial and is currently used on a trial or named patient basis in the UK. This report, however, does highlight the importance of keeping abreast of novel therapeutic developments in systemic conditions likely to be encountered by an ophthalmologist.

## Competing interests

The author(s) declare that they have no competing interests.

## Authors' contributions

(Note that this patient was treated in both Manchester and London, so doctors from both sites were involved)

DH wrote up case and collected data from Manchester notes

FEDS compared Manchester notes to London notes and contributed to write up

NGS collected data from case notes in London and contributed to write up of case

ND supervised management of the case

All authors read and approved the final manuscript.
